# Optimization of wear loss in silicon nitride (Si_3_N_4_)–hexagonal boron nitride (hBN) composite using DoE–Taguchi method

**DOI:** 10.1186/s40064-016-3379-7

**Published:** 2016-09-29

**Authors:** Sachin Ghalme, Ankush Mankar, Y. J. Bhalerao

**Affiliations:** 1 Shri Chhatrapati Shivaji Maharaj CoE, Ahmednagar, Maharashtra India; 2 Mechanical Engineering Department, Manoharbhai Patel Institute of Engineering and Technology, Gondia, Maharashtra India; 3 VM Institute of Engineering and Technology, Nagpur, India; 4 MIT Academy of Engineering, Alandi, Pune, India

**Keywords:** Silicon nitride (Si_3_N_4_), Hexagonal boron nitride (hBN), Alumina (Al_2_O_3_), Design of experiment (DoE), Taguchi technique

## Abstract

**Introduction:**

The contacting surfaces subjected to progressive loss of material known as ‘wear,’ which is unavoidable between contacting surfaces. Similar kind of phenomenon observed in the human body in various joints where sliding/rolling contact takes place in contacting parts, leading to loss of material. This is a serious issue related to replaced joint or artificial joint.

**Case description:**

Out of the various material combinations proposed for artificial joint or joint replacement Si_3_N_4_ against Al_2_O_3_ is one of in ceramic on ceramic category. Minimizing the wear loss of Si_3_N_4_ is a prime requirement to avoid aseptic loosening of artificial joint and extending life of joint.

**Discussion and evaluation:**

In this paper, an attempt has been made to investigate the wear loss behavior of Si_3_N_4_–hBN composite and evaluate the effect of hBN addition in Si_3_N_4_ to minimize the wear loss. DoE–Taguchi technique is used to plan and analyze experiments.

**Conclusion:**

Analysis of experimental results proposes 15 N load and 8 % of hBN addition in Si_3_N_4_ is optimum to minimize wear loss against alumina.

## Background

Mechanical behavior of various machine elements, such as gears, cams, wheels, rails and sealing parts are influenced by the interaction between contact elements and surfaces. Contact fatigue is a surface-pitting type failure, defined as kind of damage caused by changes in material microstructure which result in crack initiation followed by crack propagation, under the influence of time-dependent rolling/sliding contact loads (Fajdiga and Sraml 2009). Development of modern engineering application with ecological and economical aspects and development of devices operating under extreme operating conditions (high temperature, vacuum) needs some potential substitute for traditional materials. Silicon nitride (Si_3_N_4_) based ceramics presents good substitute for these conditions due to their hardness, excellent chemical, and stability under a broad range of temperature, low density, low thermal expansion and high specific stiffness (Dill 1996). In the 1950s, the interest in Si_3_N_4_ increased when it was prepared for refractory application. Biocompatibility of Si_3_N_4_ has presented scope for its use in the field of biomedical also (Neumann et al. 2004). Last 50 years research in the field of orthopedics trying to evaluate the biomaterials for hip joint replacement with improved performance in terms of extending joint life. In early days different kind of natural materials like wood, glue, rubber, tissue from living forms and manufactured materials like iron, gold, and zinc were used as biomaterials based on trial and error. Biomaterials are such materials which are intended to replace a part or function of the body in reliably economically and physiologically acceptable manner. It is estimated that approximately 250,000 knee replacements and 1 million hip replacements are carried out per year (Sculco 1995). It is expected that this number will double till 2025 as a result of aging populations worldwide and growing demand for a higher quality of life (Rakhorst and Ploeg 2008; Steven et al. 2007). The first metal-on-metal (CoCr–CoCr) total hip replacement (THR) was unsatisfactory in terms of high friction forces and high rate of wear. Titanium alloys and stainless steel are also frequently used in THR, but the main risk with use of metal alloy implants is the release of metal ions due to wear and creating a negative effect like aseptic loosening caused by adverse biological reactions due to wear products. Therefore, metal-on-ultra high molecular weight polyethylene (UHMWPE) bearing became advantages or preferable to the metal-on-metal system. A lot of literature from hip simulator studies proved improvement in wear resistance of cross-linked UHMWPE (McKellop et al. 1999; Muratoglu et al. 1999). Since from last four decades, bio-inert alumina ceramic (aluminum oxide) have presented an attractive alternative for THR bearing surface in terms of improved wear resistance and extended joint life. In late eighteenth century, the controlled implantation of bioceramic started in dental with the use of plaster of Paris or gypsum for bone filling. Ceramic bearings were first introduced as alternatives to polyethylene (PE) bearings in THR about a decade after Sir John Charnley introduced the first durable THR with a metal-PE articulation. In 1965, the first alumina (Al_2_O_3_) material dedicated for hip joint was patented, and pioneering application of bioceramic was replacing traditional metallic femoral heads of hip prostheses using high density and pure alumina. The Al_2_O_3_ and ZrO_2_ like oxide have a lengthy history in the field of hip and knee joint replacement providing a tougher bearing surface with low wear rate. With high fracture toughness and more resistant to crack propagation than alumina, Si_3_N_4_ presents an alternative to oxide ceramic with comparable wear rate (Bal et al. 2009; Cappie et al. 2010). Mode of failure in THR is related to tribology i.e. wear of cup and head. Accumulation of wear at implant leads to aseptic loosening and failure of THR. Therefore, it is desirable to reduce the generation of wear particle in the implant. Olofsson et al. (2012) conducted sliding contact wear test using pin-on-disc (PoD) tribometer with Si_3_N_4_ and CoCr disc against Si_3_N_4_ and Al_2_O_3_ ball in the presence of phosphate buffered saline (PBS) and bovine serum. Si_3_N_4_ sliding against Si_3_N_4_ showed low wear rate in both PBS and bovine serum comparable to other pairs. Xu and Kato (2000) investigated wear performance of silicon nitride sliding against itself in water showing the low coefficient of friction and low wear. The wear of silicon nitride in water occurs mainly due to the tribo-chemical dissolution of material without the release of the solid particle. Boshitskaya et al. (1996) presented that silicon nitride powder dissolve in blood serum, gastric juice, and a synthetic biochemical media at pH 7.4, suggesting the use of silicon nitride for hip joint replacement with less wear and those produced wear particles would be biodegradable. Considering orthopedic application improved the coefficient of friction and low wear rate of silicon nitride are confirmed and advantages over CoCr alloy (Rahaman et al. 2007). The ability of silicon nitride to be formulated into porous substrate and a hard bearing surface makes it best alternative in orthopedic and THR materials list. Hexagonal boron nitride is well known solid situ lubricating material with biocompatibility (Gangopadhyay et al. 1997; Saito et al. 1999; Shah et al. 2013). Anabtawi et al. (2008) evaluated the biocompatibility of boron coatings and Klepper et al. (2008) presented tribomechanical properties of thin boron coatings on cobalt alloy in an orthopedic application with no loss of coating during the test. The lubrication properties of h-boron nitride are comparable to those of phospholipids, which are the best lubricant in human (Pawlak et al. 2008). Bor Incorporation of the solid lubricant in Si_3_N_4_ can be considered to improve the tribological performance of Si_3_N_4_. Formation of an oxide of hydrated layers [H_3_BO_3_ and BN(H_2_O)_x_] has a significant effect on the tribological performance of Si_3_N_4_–BN composites, reducing the wear coefficient. Carrapichano et al. (2002) conducted sliding wear test on PoD tribometer for Si_3_N_4_–BN composite in a self-mated pair, with 10, 18 and 25 vol% of BN in Si_3_N_4_. They concluded that addition of Boron up to 10 % improved tribological properties of Si_3_N_4_ and further addition affect to mechanical properties of Si_3_N_4_. Chen et al. (2008) investigated sliding wear behavior of Si_3_N_4_–hBN composite with 0, 5, 10, 20 and 30 vol% of hBN in Si_3_N_4_ against Si_3_N_4_ using PoD tribometer. They reported that friction coefficient reduces up to 0.19 for 20 % hBN in Si_3_N_4_.

To investigate the wear behavior of Si_3_N_4_–hBN against alumina, which is not covered earlier needs experimentation using PoD tribometer. In this paper, it is attempted to study the effect of hBN addition on wear behavior of Si_3_N_4_ against Al_2_O_3_ counterface. The experimentation and analysis are done using design of experiment–Taguchi method.

## Methods

### Preparation of samples

**Fig. 1:**
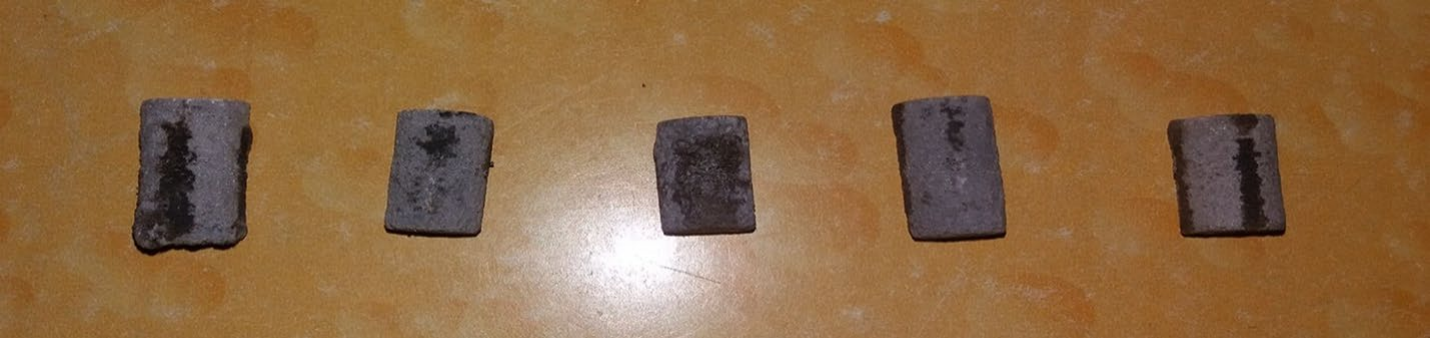
Sintered pin samples

Si_3_N_4_–hBN composites prepared with 4, 8, 12 and 16 vol% of hBN mixed in Si_3_N_4_. During preparation of pin sample, the 99 % pure powder of Si_3_N_4_ and hBN of 1-μ size (Yingkou Tanyun Chemicals Co., Ltd., China) mixed in said proportion with the help of ball mill. The mixed powder then sintered at uniaxial hot-pressing in an inert atmosphere at 30 MPa, 1600 °C and 60 min dwell time with an additive of polyvinyl alcohol into a pin of the dimension of 10 mm diameter and 15 mm long. Figure 1 shows composite pin specimens.

Tables 1 and 2 shows the corresponding density of sintered pin sample and properties of alumina disc respectively.

**Table 1:** Properties of sintered sample

Sample	1 (4 % hBN)	2 (8 % hBN)	3 (12 % hBN)	4 (16 % hBN)	5 (0 % hBN)
Density (g/cc)	1.96	1.96	1.93	1.84	2.04
Vickers hardness (MPa)	2775.88	2318.17	1741.07	907.96	7484.51

Testing at Central Glass and Ceramic Research Institute, Kolkata (India)

**Table 2:** Typical properties of alumina disc

Designation	Purity (%)	Density (g/cc)	Max service temp. (°C)	Avg. surface roughness Ra (μm)
Alumina (Al_2_O_3_)	99.8	3.90	1800	1.791

### Experimental setup

The wear tests were conducted on Ducom TR-20LE-PMH400 pin on disc tribometer with following major specifications:

1. Specimen pin size: 3, 6, 8, 10 and 12 mm diameter, 25–30 mm long.
2. Wear disc size: 165 mm diameter and 8 mm thick.
3. Disc rotation: min. 200 rpm, max. 2000 rpm.
4. Normal load: min. 5 N, max. 200 N.
5. Temperature: min. ambient, max. 400 °C.

Tests were performed according to ASTM F732 standards (2011) with following test parameters:

1. Si_3_N_4_–hBN composite pin and Al_2_O_3_ disc counterface.
2. The speed of disc: 200 rpm.
3. Test duration: 20 min.
4. Without lubricant at atmospheric conditions.

Si_3_N_4_–hBN and alumina both are a biocompatible material, but in the primary stage of material investigation sliding wear test using a pin on the disc is appropriate (Olofsson et al. 2012). So, we conducted tests in a dry environment with a lubricant to check its wear performance.

## Methodology: Taguchi Method

The design of experiments (DOE) is a tool for planning, designing and analyzing the experiments so that valid and objective conclusions can be drawn effectively and efficiently from results of the experiments (Antony 2008). Taguchi method is a form of DOE developed by Genichi Taguchi used for efficient planning and conducting experiments to analyze how different parameters affect the mean and variance of a process performance characteristic. Taguchi proposed a special design of orthogonal arrays to study all parameters at their corresponding levels with a small number of experiments only. The results of the experiments are further transformed into a signal-to-noise (S/N) ratio. The S/N ratio is a measure of quality characteristics deviating from or nearing to the desired values. Ghalme et al. (2013) have implemented DOE–Taguchi technique to investigate the effect of surface roughness and lubricant viscosity on the coefficient of friction in rolling contact. Analysis of result presented strong interaction of surface roughness and lubricant viscosity on the coefficient of friction in rolling contact. Patnaik et al. (2009) implemented DOE–Taguchi design technique to evaluate the tribo-performance of polyester hybrid composites. The result presented that glass-polyester composite without any filler suffers greater erosion loss than the hybrid composite with alumina filling. Lastly the results were optimized using a genetic algorithm. One of the best thing with the use of Taguchi method is the use of the experimental design, involving the use of orthogonal arrays to organize the parameters affecting the process and the levels at which they should be varied. It helps to determine which factors affect product quality with a minimum amount of experimentation, thus saving time and resources. The conclusions drawn from a minimum number of experiments are valid over the entire experimental region spanned by the control factors and their levels (Phadke 1989). Ghalme et al. (2016) demonstrated the use of DoE–Taguchi method in parameter optimization of milling of glass fiber reinforced plastic (GFRP) with only nine number of experiments and found that speed and depth of cut are more significant during milling of GFRP. The parameters/factors and their corresponding levels selected for the experiments as shown in Table 3.

**Table 3:** Designed experimental factors and levels

Factors	Level 1	Level 2	Level 3	Level 4	Level 5
Load (N)	5	10	15	20	25
% hBN	4	8	12	16	0

A general procedure for optimizing any process parameters proposed by Taguchi (Muratoglu et al. 1999) involves following steps:

- Identification of the quality characteristic to be optimized.
- Identification of the noise factors and test conditions.
- Identification of the control factors and their corresponding levels.
- Designing the experimental layout and defining the data analysis procedure.
- Conducting the experiments.
- Analyzing the data and determining the optimum levels of control factors.
- Predicting the performance at these levels.

Load and percentage of hBN are two factors selected at five levels as shown in Table 3. Therefore using relation:

1 $$level^{Factor} = 5^{2} = 25$$

L25 orthogonal array with 25 number of experiment selected for conduction of experiments. The orthogonal array provides a set of well-planned experiment with the minimum number.

### Results and signal to noise (S/N) ratio

Experiments were conducted on PoD tribometer with two input parameters and wear volume loss of a sample as output. Wear volume loss calculated for covered sliding distance with 200 rpm and 20 min duration. Table 4 shows results of all 25 experiments (each experiment repeated four times) along with transformed into signal to noise (S/N) ratio. Taguchi’s S/N ratios, which are logarithmic, function of desired output ad serves as an objective function for optimization. The standard S/N ratios used are: smaller is better (SB), nominal is better (NB), and higher is better (HB). In this study, S/N ratio with SB was used for wear volume loss. SB S/N ratio is calculated as follow:

2 $$(S/N)_{SB} = - 10log_{10} \left( {\left( {y_{1}^{2} + y_{2}^{2} + y_{3}^{2} + \cdots } \right)/n} \right)$$

where *y*_1_, *y*_2_ and so on = experimental results/observation, n = number of experiments (*y*_*i*_).

**Table 4:** Results for wear volume loss (WVL) and S/N ratio

Expt. no.	Load (N)	% hBN	Avg. WVL (mm^3^/m)	S/N ratio (dB)
1	5	4	0.3022	10.3941
2	5	8	0.199	14.0229
3	5	12	0.2444	12.2379
4	5	16	0.0337	29.4319
5	5	0	0.5055	5.9263
6	10	4	0.2007	13.9499
7	10	8	0.0156	36.1264
8	10	12	0.1437	16.8466
9	10	16	0.0205	33.7861
10	10	0	1.0867	−0.7222
11	15	4	0.314	10.0614
12	15	8	0.0111	39.0935
13	15	12	0.1029	19.7516
14	15	16	0.0953	20.4163
15	15	0	1.3473	−2.5893
16	20	4	0.2063	13.7101
17	20	8	0.5002	6.0166
18	20	12	2.2799	−7.1583
19	20	16	0.2035	13.8287
20	20	0	0.3169	9.9816
21	25	4	0.5142	5.7774
22	25	8	2.112	−6.4856
23	25	12	0.4144	7.6516
24	25	16	0.1551	16.1877
25	25	0	4.1178	−12.2933

The maximization of S/N ratio maximizes the desirable characteristic against noise factors. The desirable characteristic of this work is the minimization of wear volume loss. Observation of S/N ratio gives an optimal combination of input parameters for required output characteristic. From table expt. 12 offers an optimal combination of 15 N load and 8 % hBN for minimum wear volume loss of 0.011 mm^3^/m with corresponding maximum S/N ratio of 39.09354042 dB.

### Response plot

**Fig. 2:**
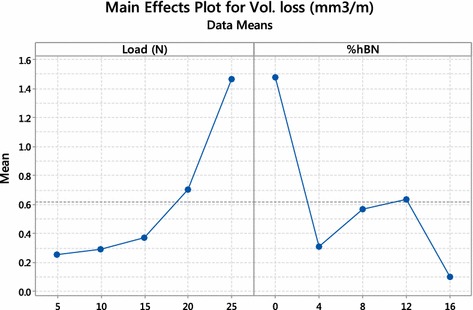
Main effect plot for wear volume loss

**Fig. 3:**
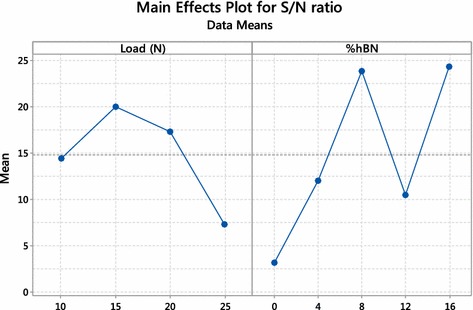
Main effect plot for S/N ratio

**Fig. 4:**
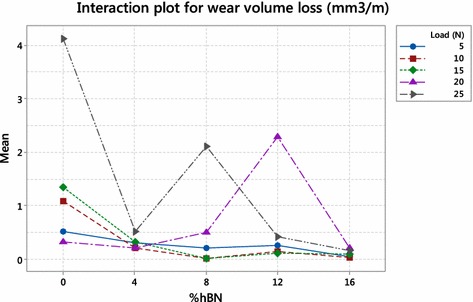
Interaction plot for wear volume loss

**Fig. 5:**
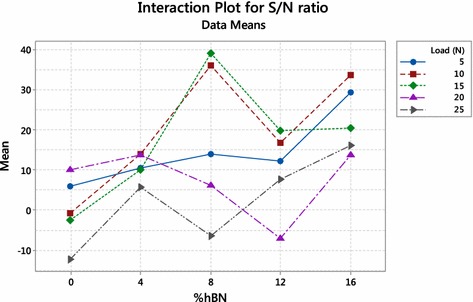
Interaction plot for S/N ratio

**Fig. 6:**
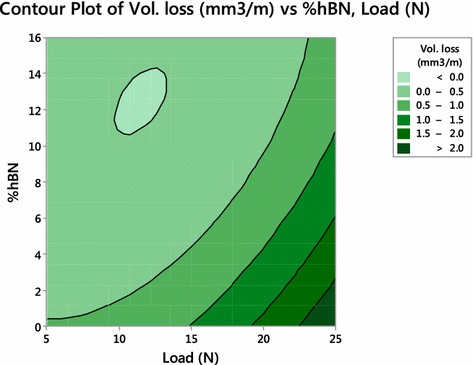
Contour plot of wear volume loss

Figures 2, 3, 4, 5 and 6 represents various response plot for experiments prepared with MINITAB 17. Main effect plot represents the effect of process variable on the response. Figure 2 shows the effect of individual load and percentage of hBN on wear volume loss.

Figure 3 shows corresponding S/N ratio of the individual value of parameter i.e. load and percentage of hBN, indicating a maximum value of S/N ratio at 15 N load and 8 % hBN.

Interaction plot represents interaction effect of two variables on the response. Figure 4 shows interaction effect of load and percentage of hBN on wear volume loss. From interaction plot, it is clear that all lines interact with each other representing strong interaction between load and percentage of hBN on wear volume loss. The minimum mean value of wear volume loss is also observed at 8 % hBN and all loading condition except 25 N. Figure 5 represents the variation of S/N ratio against interaction of load and percentage of hBN, showing maximum S/N ratio at a combination of 15 N load and 8 % hBN.

Contour plot represents control parameters on x and y-scale and response variable as a contour. It represents the clear variation of response variable against control variables. Figure 6 shows contour plot for wear volume loss against load and percentage of hBN. From observation of contour plot also, it is clear that wear loss is interaction effect of load and % hBN.

### ANOVA

Analysis of variance (ANOVA) is performed to investigate the effect or percentage contribution of individual control factor on the response variable. Table 5 shows ANOVA for wear volume loss.

**Table 5:** ANOVA table for wear volume loss

Source	DF	Seq SS	Contribution (%)	Adj MS
Load (N)	4	5.075	23.51	1.2627
% hBN	4	5.501	25.48	1.3752
Load (N) % hBN	16	11.012	51.01	0.6882
Total	24	21.588	100	

In ANOVA table (Ghalme et al. 2016):

- The degree of freedom (DF) is a measure of amount independent information available from given set of data. DF for concerning factor is one less than the number of levels.
- The sequential or adjusted sum of squares (Seq SS/Adj SS) of factor measures the variability in data contributed by that factor. Total SS is SS of an individual factor and SS of error.
  3 $$Seq\,SS_{factor} = \sum {n_{i} \left( {\bar{y}_{i} - \bar{y}} \right)^{2} }$$
  4 $$Seq\,SS_{error} = \sum\limits_{i} {\sum\limits_{j} {\left( {y_{ij} - \bar{y}_{i} } \right)^{2} } }$$
  5 $$Seq\,SS_{total} = \sum\limits_{i} {\sum\limits_{j} {\left( {y_{ij} - \bar{y}} \right)^{2} } }$$
  where $\bar{y}_{i}$ mean of all observations at *i*th factor level, $\bar{y}$ mean of all observations, *y*_*ij*_ value of *j*th observation at the *i*th factor level, *n*_*i*_ number of observations for the *i*th factor level.
-  Adjusted mean squares (Adj MS) or variance is Seq SS divided by DF.
-  Percentage contribution signifies individual contribution of a factor on the mean response. It is calculated by:
  6 $$\% \,contribution = \frac{{Seq\,SS_{factor} }}{{Seq\,SS_{total} }} \times 100$$

From the table, it is clear that percentage hBN has 25.48 % contribution and load has 23.51 % contribution to wear volume loss of composite. While combined load and percentage of hBN has the major contribution of 51.01 % to wear volume loss.

### Confirmation experiment

From results, S/N ratio, response plot, and ANOVA it is evident that percentage of hBN has a significant effect on wear volume loss, and combined load and percentage of hBN have a major role in wear of composite. The analysis also presents 8 % hBN as optimum to minimize wear volume loss of silicon nitride–hBN composite against alumina counterface. To validate these conclusions, confirmation experiments were conducted at different loading conditions and 8 % hBN in silicon nitride. Table 6 shows results of confirmation experiments. The results also present, that there is no significant increase in wear volume loss with an increase in load.

**Table 6:** Experimental conditions and results

Expt. no.	Load (N)	% hBN	Expt. result-WVL (mm^3^/m)
1	50	8	4.01204
2	100	8	5.0456
3	150	8	5.0498

## Results and discussion

In this article, five samples of Si_3_N_4_–hBN are evaluated against alumina for its wear performance. The results of the experiments along with S/N ratios are presented in Table 4. DoE–Taguchi method is a suitable technique in the field of process parameter optimization and experimental analysis (Ghalme et al. 2013, 2016; Patnaik et al. 2009; Phadke 1989). Using this technique effect of load and % hBN addition on wear performance of Si_3_N_4_ is evaluated against alumina counterface. Carrapichano et al. (2002) concluded that 10 % addition of boron could improve the wear performance of Si_3_N_4_ in a self-mated pair. But they have not considered the effect of the load. When we are proposing Si_3_N_4_–hBN composite as an alternative for hip/knee joint replacement (Neumann et al. 2004; Boshitskaya et al. 1996; Gangopadhyay et al. 1997; Saito et al. 1999), it is clear that an amount weight of a person changes from person to person. In this work, we tried to evaluate the combined effect of load and % of hBNon wear loss of silicon nitride. We found that 15 N load and 8 % hBN in Si_3_N_4_ is suitable to minimize its wear loss against alumina counterface.

## Conclusion

1. The results of Taguchi analysis and confirmation experiments presents the optimum proportion of 8 % hBN in Si_3_N_4_ for minimization of wear loss against alumina counter-face. Thus, Taguchi method not only useful to plan experiments but helps to analyze the results of the experiments.
2. From experimental results, S/N ratio and ANOVA it is clear that wear performance is a function of load and % of hBN. From this experimental analysis 15 N load and 8 % hBN is optimum to minimize wear volume loss of Si_3_N_4_ against alumina counter-face. It indicates load and % hBN has combined effect on wear volume loss.
3. From confirmation experiments also it is clear that for 8 % hBN in Si_3_N_4_, wear volume loss increase with an increase in load. It also signifies that wear loss in Si_3_N_4_ is a function of load and % hBN addition.
